# Comparison of the effect of different medicaments on surface reproduction of two commercially available Polyvinyl Siloxane impression materials - An Invitro Study

**DOI:** 10.4317/jced.51134

**Published:** 2013-07-01

**Authors:** Rina Singh, Jagjit Singh, Ramandeep S. Gambhir, Ramanpreet Singh, Sonia Nanda

**Affiliations:** 1 BDS, MDS. Sr. Lecturer, Dept. of Prosthodontics. Gian Sagar Dental College and Hospital, Rajpura, Punjab. India; 2 BDS, MDS. Professor, Dept. of Periodontics. Gian Sagar Dental College and Hospital, Rajpura, Punjab. India; 3 BDS, MDS, MPH. Sr Lecturer, Dept. of Public Health Dentistry. Gian Sagar Dental College and Hospital, Rajpura, Punjab. India; 4 BDS, MDS. Reader, Dept. of Prosthodontics. Gian Sagar Dental College and Hospital, Rajpura, Punjab. India; 5 BDS, MDS. Sr. Lecturer, Dept. of Prosthodontics, PGIMER, Rohtak, Haryana. India

## Abstract

Objective:To determine the effect of different retraction cord medicaments on surface detail reproduction of polyvinyl siloxane impression materials and compare this effect on any two brands of commercially available polyvinyl siloxane impression materials. Material and methods: Four stainless steel dies were made according to ADA specification no.19. Three dies were treated with aluminium chloride (5%), ferric sulphate (13.3%) and epinephrine (0.1%) while the fourth one was left untreated to serve as control. Two impression materials (Dentsply and 3M ESPE) were used. Results: All the three medicaments adversely affected the surface detail reproduction of both the brands of the polyvinyl siloxane impression materials. These effects were statistically significant as compared to untreated control. The impressions of 3M ESPE brand have shown better surface detail reproduction as compared to Dentsply impression material. Conclusion: Surface detail reproduction of the polyvinyl siloxane impression materials is adversely affected by the retraction cord medicaments. The presence of moisture or any traces of the medicaments should be removed from the tooth surface to provide a dry field for the correct reproduction of the surface detail of these materials.

** Key words:**Polyvinyl Siloxane, retraction cord medicaments, surface detail reproduction.

## Introduction

Accurate reproduction of prepared tooth or edentulous arch is of critical importance in the fabrication of fixed or removable restorations. Inaccuracies in replication process will ultimately have an adverse effect on adaptation and fit of the prosthesis (1). Amongst all the other elastomeric impression materials, the most commonly used impression material is polyvinyl siloxane. The popularity of polyvinyl siloxane impression material is attributed to several characteristics including the dimensional stability, dimensional accuracy, and excellent elastic recovery, ease of manipulation, superior electroplating qualities and good shelf life (1,2).

Apart from the above-mentioned advantages, the polyvinyl siloxane impression materials have few serious limitations. Because of their extremely hydrophobic nature, there should not be any moisture in the gingival crevice and it is most difficult to pour the cast. Many researchers have tried to make it hydrophilic by incorporating surfactants into the material, which reduce the contact angle, improve the wettability and simplify the pouring of gypsum models (3). Moreover, the polymerization of hydrophilic polyvinyl siloxane impression material is inhibited by sulfur compounds which are contained in latex gloves, rubber dam sheets and some surface containing retraction cord medicaments such as aluminum sulfate and ferric sulfate (4,5,6).

Tissue displacement is commonly needed to obtain adequate access to the prepared tooth to expose all the necessary surfaces, both prepared and unprepared. An impression made should record not only the finish line and all the prepared surfaces of the tooth, but also an area of unprepared tooth surface immediately beyond the finish line (7). This facilitates the dentist and laboratory technician to identify the contour of tooth and all prepared surfaces. Retraction cord and medicament incorporated in the cord cause transient ischemia and displaces the marginal gingiva laterally to expose the finish line and tooth surface. If impression does not reproduce this critical area where tooth and future restoration meet, fabricating the restoration with marginal adaptation to finish line and proper contour is not possible. This ultimately results in the failure of the prosthesis (7).

As sulfur containing medicaments inhibit the polymerization of polyvinyl siloxane impression material; it results in rippled surface and affects the surface detail reproduction (8). Similar to sulfur containing medicaments, many other medicaments used with retraction cord may come in contact with impression material and may affect its surface detail reproduction (9,10). Hence this study was planned to find out whether these medicaments affect the detailed reproduction of the impression material and to compare the effect on two commercially available polyvinyl siloxane impression materials.

## Material and Methods

This prospective, in vitro study was conducted in the Department of Prosthodontics involving Implantology and Department of Oral Pathology, Bapuji Dental College and Hospital to determine the effect of three retraction cord medicaments on surface detail reproduction of polyvinyl siloxane impression materials.

Three retraction cord medicaments containing aluminum chloride 5%, epinephrine 0.1 % and ferric sulfate 13.3% were utilized in the study. A medium bodied Type 2 hydrophilic polyvinyl siloxane impression material (Aquasil ultra Monophase. Lot No 040727) was obtained from Dentsply, India private limited. Another brand of medium bodied polyvinyl siloxane was obtained from 3M ESPE.

**Figure 1:**
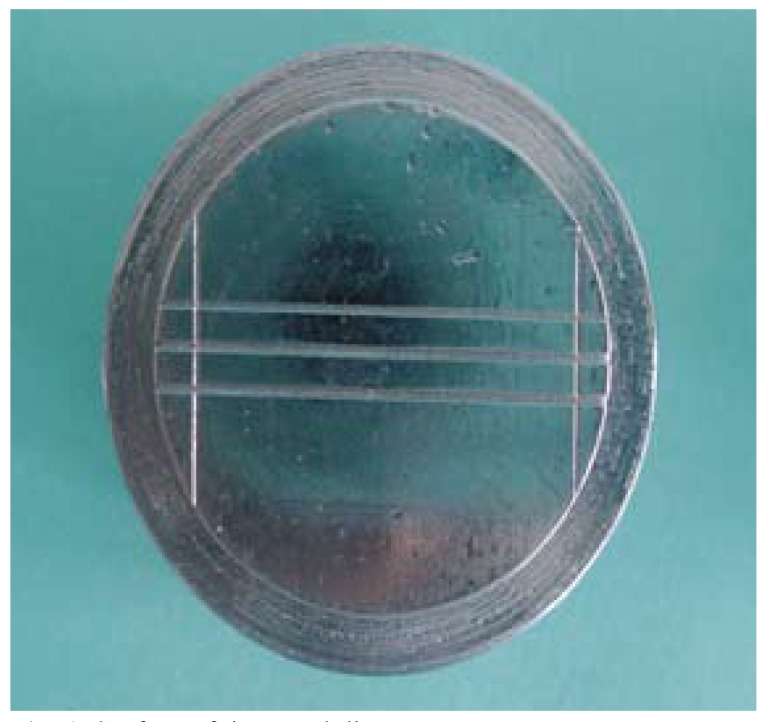
Surface of the metal die.

Four standardized stainless steel dies (as described in ADA specification No. 19) were used for the study. The dies were fabricated at Bapuji Institute of Engineering and Technology. The surface of each die was having following specifications (Fig. 1).

● Two vertical lines were scored at a distance of 25 mm between them.

● Three horizontal lines were scored and numbered 1, 2 & 3. They were separated from each other by 5mm. Each line was having the width 160µ. The height on top of the die was 2mm over which a mold of 5mm was made to support the impression material and the resultant impression material thickness obtained was thus 3mm.

- Impression Procedure and Preparation of Specimen

For making any new impression, each time the dies were cleaned in ultrasonic cleaner using distilled water to remove any residues of medicament and impression material. The die was dried with compressed air. Care was taken to protect the surface of the die to avoid any contamination. Impressions were made using an auto mixing impression gun of Hereaus Kulzer and prepackaged cartridges of Aquasil monophase impression material and 3M ESPE material. An acrylic mold 5mm high was placed on the die to support impression material. The material was loaded into a fine tipped impression syringe and applied to lined areas of die. Care was taken to ensure that tip was in contact with the metal die. The impression material was pushed ahead of syringe tip. A polyethylene sheet followed by a flat glass plate was placed on top of the mold to keep polyvinyl siloxane within the mold. The die was transferred to thermostatically controlled water bath. To ensure that die did not move, 500 gm weight was placed on top of the flat glass plate. Water bath maintained at temperature 32° ± 1 °c (to simulate oral conditions) was used in accordance with ADA specification No. 19.

- Collection of specimens

The entire assembly i.e. dies; polyethylene sheet, metal plate, flat glass plate, and weight were removed from the water bath after 13 minutes. The impressions were allowed to set for five minutes longer than the manufacturers recommended minimal removal time indicated in ADA specification No.19 for lab testing. The mold and die were then separated from the impression and impression was numbered on back with a marker. Fifteen sample impressions were made of metal dies treated with each of three retraction cord medicaments and for each brand for a total of ninety specimens. Fifteen additional impressions of untreated dies were also made for each impression material and these served as controls.

- Evaluation of surface detail reproduction

Surface detail reproduction was evaluated one hour after removal of the impression from the water bath. Each horizontal line was evaluated under the stereomicroscope at 10 magnification. The surface detail reproduction was considered acceptable, if two or three of the horizontal lines were reproduced continuously and well defined for 25 mm between the cross lines. The reproduction of the line was considered unacceptable if any part of the line was indistinct, e.g. appeared melted or flattened, or the borders of the line were fuzzy or blurred. Even if there is any pooling of liquid around the edges of the line, the line was considered unacceptable. An impression was considered successful if at least two of the three lines were accurately reproduced.

The impressions obtained from the die were divided into four groups and evaluation of the surface reproduction of the horizontal lines was done with both the impression materials microscopically-

Group I: Impressions of 5% aluminium chloride treated dies 

Group II: Impressions of 13.3% Ferric Sulphate treated dies

Group III: Impressions of 0.1% Epinephrine treated dies 

Group IV: Impressions of untreated die 

- Statistical Analysis

The findings for all the four groups were tabulated and statistically analyzed. To determine the effect of medicaments and compare the two impression materials chi square and fisher’s exact test were used. A p value of less than 0.05 was considered as significant.

## Results

The aim of the study was to determine the effect of three retraction cord medicaments (5% aluminium chloride, 13.3% ferric sulphate and 0.1% epinephrine) on the two brands of medium body polyvinyl siloxane impression materials (Aquasil- Dentsply and Imprint II- 3M ESPE).

**Figure 2:**
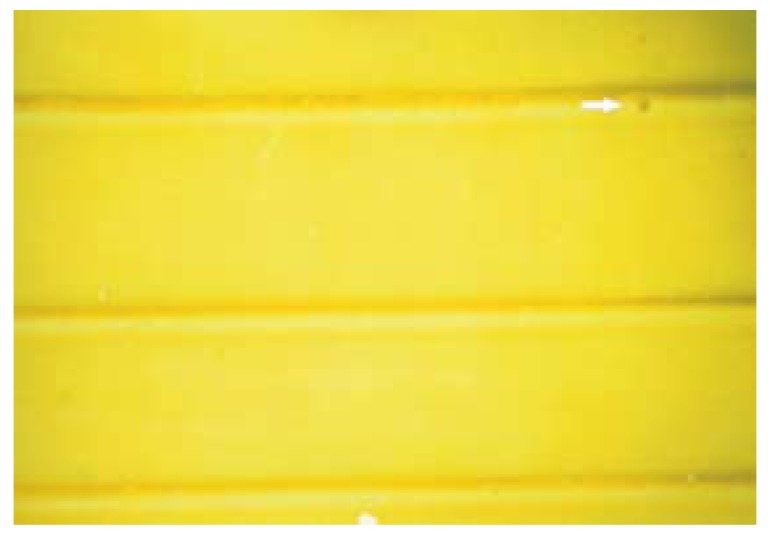
Satisfactory impression.

**Figure 3:**
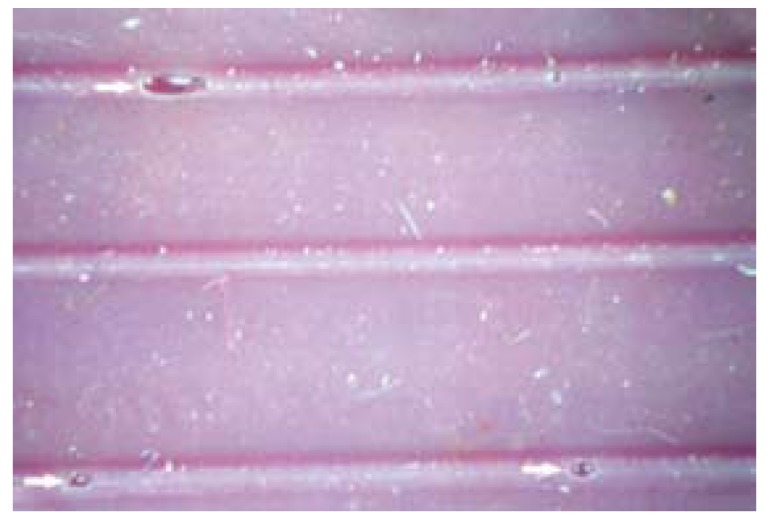
Unsatisfactory Impression.

The no. of satisfactory and unsatisfactory impressions of all the four groups was evaluated with both the impression materials (Fig. 2,3). The number of impressions showing satisfactory surface detail reproduction of Groups I, II, III and IV were 4, 4, 6 and 12 respectively and the number of impressions showing unsatisfactory surface detail reproduction for Groups I, II, III and IV were 11, 11, 9 and 3 respectively with Dentsply impression material ( Table 1).

**Table 1:**
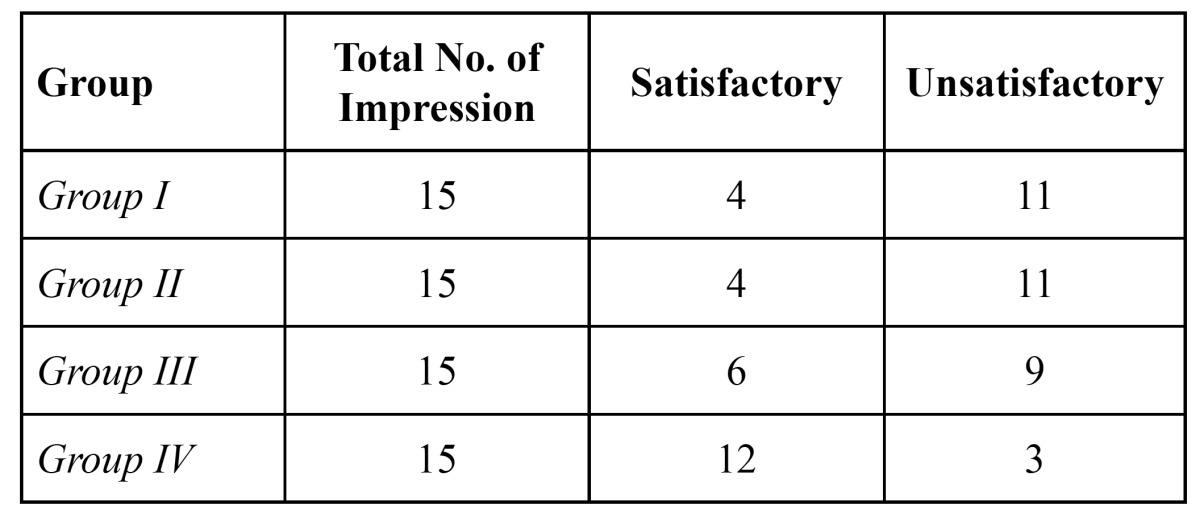
Number of impressions showing satisfactory and unsatisfactory surface detail reproduction for Dentsply impression material.

 Table 2 shows the number of satisfactory and unsatisfactory impressions of all the four groups when 3M ESPE impression material was used. The number of impressions showing satisfactory surface detail reproduction of Groups I, II, III and IV were 11, 4, 8 and 13 respectively. The number of impressions showing unsatisfactory surface detail reproduction for Groups I, II, III and IV were 5, 11, 7 and 2 respectively.

**Table 2:**
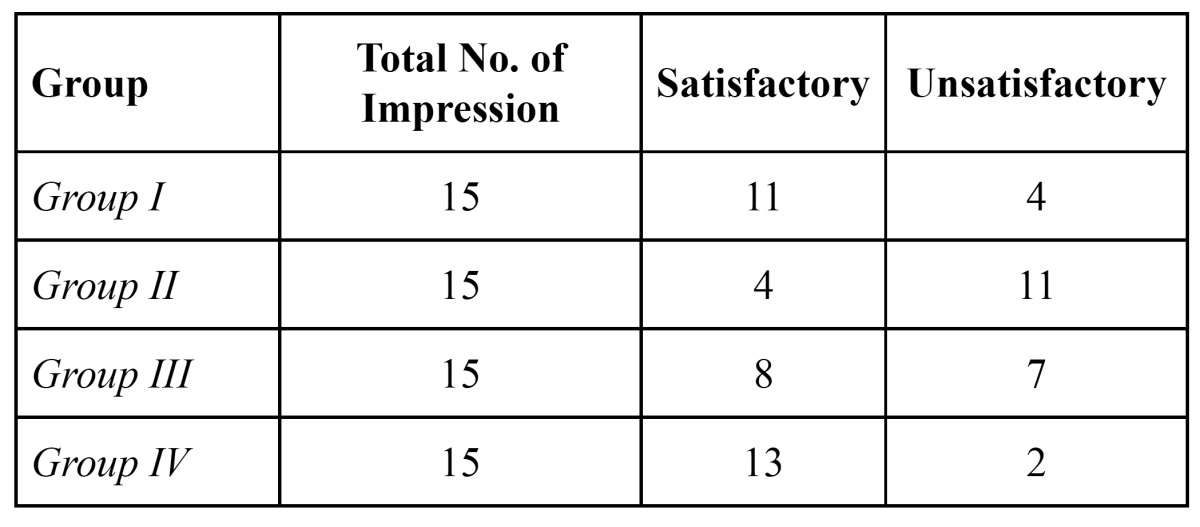
Number of impressions showing satisfactory and unsatisfactory surface detail reproduction for 3M ESPE (II) impression material.

 Table 3 shows the statistical significance and comparison between the two bands (I and II) of impression materials. Fisher’s Exact Test and Chi Square Test were used to compare the surface detail reproduction of I and II impression materials. The table indicates a statistically significant result in group I when the two materials are compared. The chi square value was 6.53 and P<0.05 showing the statistical significance. When I and II were compared for Group II no statistical significant difference was seen (P> 0.05). Similarly in Group III and IV no statistically significant difference was seen when both the brands of impression materials were compared (P>0.05). The table clearly indicates that both the brands of the polyvinyl siloxane impression material have been affected by the retraction cord medicaments. Group I has shown statistically significant results and though in other groups statistically non significant results have been seen, 3M ESPE impression material has shown better surface detail reproduction as compared to Dentsply impression material.

**Table 3:**
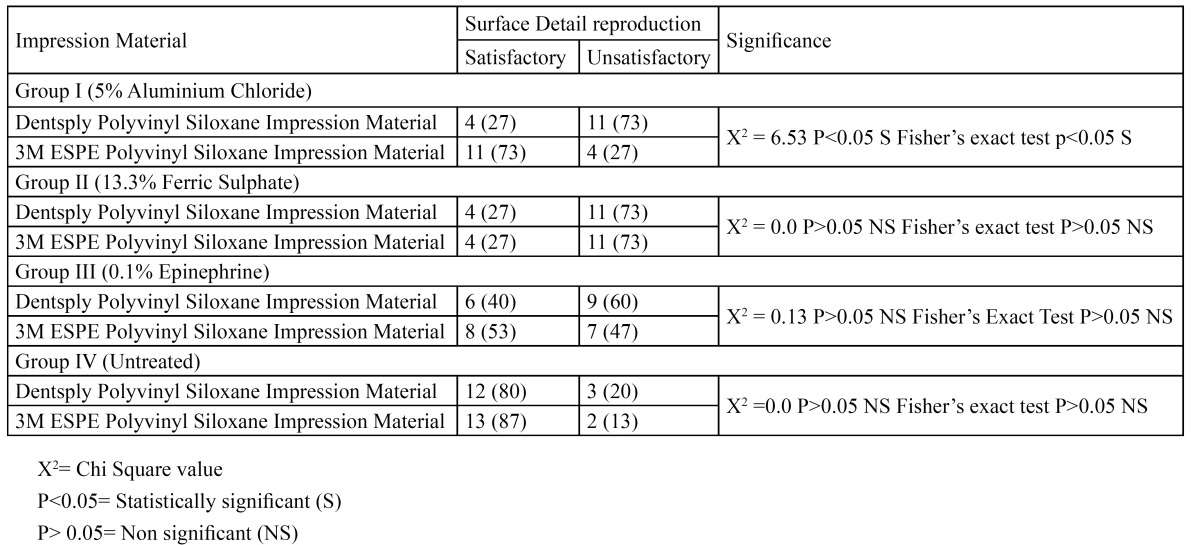
Statistical significance and overall comparison of the surface reproduction of Dentsply (I) and 3M ESPE (II) impression materials.

## Discussion

Polyvinyl siloxane materials have gained wide popularity because of their excellent physical properties including dimensional accuracy, dimensional stability, and elastic recovery. Ease of handling, ability to pour multiple casts from a single impression and good surface reproduction has also contributed to their wide acceptance (1,11,12).

Though the polyvinyl siloxane impression materials have good surface detail reproducibility, studies have reported that the polymerization of polyvinyl siloxane is inhibited when they come in contact with sulphur during polymerization, which in turn also affects the surface detail reproduction. Sulphur is contained in latex gloves, rubber dam sheet and few haemostatic agents (4-6,13,14). Studies conducted on surface detail reproduction of polyvinyl siloxane under dry, moist and wet conditions have shown that surface detail reproduction is affected under moist and wet conditions (1,15).

The apical and lateral displacement of gingiva for exposing finish line is a prerequisite for making accurate dental impressions. Currently the impregnated cords with medicaments are most commonly used for gingival retraction (16). During gingival retraction procedures, impregnated cords placed in gingival sulcus release the medicament, which in turn affects the surface detail reproduction of the polyvinyl siloxane impression materials (2,8).

Studies have shown conflicting reports regarding the effect of retraction cord medicaments on the surface detail reproduction of poly vinyl siloxane impression materials. The results showed no inhibition of polymerization (2,8).

Another study conducted by O’Mahony et al (2) was conducted to evaluate the effect of retraction cord medicaments on the surface detail reproduction of polyvinyl siloxane impressions. The results of the study showed that the retraction cord medicaments aluminium chloride, ferric sulphate and ferric subsulfate have affected the surface detail reproduction.

The results of the present study are in accordance with the above mentioned study where the authors mentioned that the surface detail reproduction was affected by retraction cord medicaments like aluminium chloride, ferric sulphate and ferric sub sulphate.

Petrie CS et al (1) and Johnson GH et al (15) conducted experiments where it was shown that the presence of moisture affected the surface detail reproduction of the polyvinyl siloxane impression material. According to recent evidence from another study, polyether material is more likely to produce impressions with superior detail reproduction as compared to polyvinyl siloxane in the presence of moisture (17). In the present study, any undetected moisture from the surface of the die was dried with compressed air. Drying with compressed air alone will not completely eliminate the traces of medicaments and this is similar to a clinical situation, where clinician dried the tooth with compressed air but fails to rinse the medicament off the tooth and gingival sulcus before making an impression.

Browning GC et al (18) conducted a study to screen different methods to remove latex gloves contaminants from tooth and gingival surface before making impression. The authors recommended decontamination with a tooth brush or pumice to remove residues or contaminants of retraction cord medicaments. To prevent the effect of moisture content the area should be dried with compressed air before making a poly vinyl siloxane impression (1,15). Proper handling of the impression material is recommended to ensure proper results (19).

Another study conducted to evaluate the effects of sulfur-based hemostatic agents and gingival retraction cords handled with latex gloves on the polymerization of polyvinyl siloxane impression materials did not show any changes in polymerization of the impression material (20).

## Conclusion

Within the limitations of the study the following conclusion can be drawn from the present study-

1. All the three medicaments (aluminium chloride, ferric sulphate and epinephrine) affect the surface detail reproduction of the polyvinyl siloxane impression materials.

2. As compared to Dentsply impression material the 3M ESPE medium body impression material has shown better surface detail reproduction.

3. The impression making is highly technique sensitive and thus before making a polyvinyl impression, the traces of retraction cord medicaments should be removed from the tooth surface and the area should be dried so that no moisture is present.
